# Experimental observation of counter-intuitive features of photonic bunching

**DOI:** 10.1038/s41377-026-02250-4

**Published:** 2026-06-29

**Authors:** Giovanni Rodari, Carlos Fernandes, Eugenio Caruccio, Alessia Suprano, Francesco Hoch, Taira Giordani, Gonzalo Carvacho, Riccardo Albiero, Niki Di Giano, Giacomo Corrielli, Francesco Ceccarelli, Roberto Osellame, Daniel J. Brod, Leonardo Novo, Nicolò Spagnolo, Ernesto F. Galvão, Fabio Sciarrino

**Affiliations:** 1 Dipartimento di Fisica, Sapienza Università di Roma, Roma, Italy; 2 International Iberian Nanotechnology Laboratory (INL), Braga, Portugal; 3 Istituto di Fotonica e Nanotecnologie, Consiglio Nazionale delle Ricerche (IFN-CNR), Milano, Italy; 4 Dipartimento di Fisica, Politecnico di Milano, Milano, Italy; 5 Instituto de Física, Universidade Federal Fluminense, Niterói – RJ, Brazil

**Keywords:** Quantum optics, Single photons and quantum effects

## Abstract

Bosonic bunching is a term used to describe the well-known tendency of bosons to bunch together, and which differentiates their behavior from that of fermions or classical particles. However, in some situations, perfectly indistinguishable bosons may counter-intuitively bunch less than classical, distinguishable particles. Here, we report two such counter-intuitive multiphoton bunching effects observed with three photons in a three-mode balanced photonic Fourier interferometer. In this setting, we show that indistinguishable photons actually minimize the probability of bunching. We also show that any non-trivial value of the three-photon collective photonic phase leads to a decreased probability of all photons ending up in the same mode, even as we increase pairwise indistinguishability. Our experiments feature engineering of partial indistinguishability scenarios using both the time and the polarization photonic degrees of freedom, and a polarization-transparent 8-mode tunable interferometer with a quantum-dot source of single photons. Besides the foundational understanding, the observation of these counter-intuitive phenomena open news perspective in devising more efficient ways of routing photons for advantage in metrology and quantum computation.

## Introduction

A fundamental consequence of bosonic statistics is the tendency for bosons to occupy the same state, leading to physical phenomena like Bose-Einstein condensation^1^ or the Hong-Ou-Mandel effect^2,3^. In the latter, two identical photons entering different input arms of a balanced beam splitter always exit via the same output arm, due to destructive interference between the two indistinguishable paths that make the photons exit from different arms. It is well known that any distinguishability between the input photons (e.g., in the frequency or polarization degrees of freedom) provides which-path information, consequently degrading quantum interference effects and resulting in a lower probability for bosons to bunch. Thus, the common intuitive behavior is that reducing the degree of indistinguishability between multiphoton states provides a monotonic reduction in bunching effects.

In multiphoton interference experiments, several ways to quantify boson bunching have been proposed^4–6^, and the precise connection between bosonic bunching and the distinguishability of the photons is still poorly understood. While it is generally predicted and experimentally observed that any amount of distinguishability degrades boson bunching effects^4–8^, recent counterexamples have been found. In Refs. ^9,10^ it was theoretically shown that multimode bunching probabilities, i.e., the probability for all photons to coalesce in a subset of output modes, are not always maximized by perfectly indistinguishable photons. Such anomalous behavior of boson bunching was predicted to occur only in interference experiments with 7 or more photons, leaving open the question of whether other such counter-intuitive features of boson bunching can be observed in smaller experiments.

In this work, we study the role of photon indistinguishability in two natural measures of boson bunching, thus observing their counter-intuitive behavior in three-photon interference experiments. The first measure of boson bunching we consider, which we simply refer to as bunching probability, is the probability of observing outcomes with two or more photons in the same output mode. This is also referred to as the probability of collisions in Boson Sampling experiments and, when averaged over random interferometers, it is known to be larger for indistinguishable bosons than for distinguishable particles^11,12^. We demonstrate that this average behavior is not always valid. Focusing on the balanced tritter interferometer, which has already been used as a testbed for previous observations on the non-monotonicity of multiphoton bunching^13,14^, we observe experimentally not only that distinguishability can increase the bunching probability but that this quantity is, in fact, *minimized* if the photons are fully indistinguishable.

The second measure of boson bunching we consider is the full bunching probability, defined as the probability that all photons exit the interferometer in a given output port. It is known that indistinguishable photons have an exponential enhancement of full bunching in comparison with distinguishable particles^4,15^. While other output configurations are known to have a non-monotonic behavior of the corresponding probability with respect to the pairwise distinguishability of the photons^7,8,13,16,17^, full bunching probabilities are widely assumed to decrease monotonically with pairwise distinguishability, a claim that is supported by experimental observations^7,8^. We explicitly show that this, in fact, is only true in restricted set-ups, where inner products between the functions describing the internal degrees of freedom of each input photon (frequency, polarization, etc.) are given by real, non-negative values. In full generality, the relational properties of the input quantum states are described by complex Gram matrices^18^. The complex phases needed to describe such matrices can be related to many different non-classical effects^18–20^. In the context of multiphoton interference, they are referred to as collective photonic phases^21^ and only recently have they been measured in three- and four-photon experiments^14,22–24^. In our work, we unveil the role of collective photonic phases in the phenomenon of boson bunching, showing how they non-trivially affect the interference between paths that lead to all photons exiting in the same output mode. As a striking consequence of this, we experimentally demonstrate situations where full bunching probabilities can increase even though the pairwise distinguishability between the photons is decreased. In particular, we experimentally observe such counter-intuitive features in an advanced photonic platform based on a quantum-dot source and an integrated fully-reconfigurable universal and polarization-independent interferometer fabricated via the femtosecond laser micromachining technology. This is performed by exploiting the peculiar property of the employed interferometer, which enables the use of multiple internal degrees of freedom to prepare classes of states suitable for the analysis of the role of collective phases in multiphoton bunching properties.

## Results

### Theoretical overview about multiphoton indistinguishability and bunching effects

The set-up we consider consists of an *n*-mode general linear-optical interferometer, with one single photon per input mode. The interferometer is described by a unitary *n* × *n* matrix *U*, which maps input creation operators to output creation operators. The indistinguishability of the photons is completely described by a Gram matrix of inner products: ${G}_{i,j}=\langle {\psi }_{i}| {\psi }_{j}\rangle$, where $| {\psi }_{i}\rangle$ is the spectral function describing all the internal degrees of freedom of the photon entering input port *i*^15^. This Gram matrix can be written only in terms of physically relevant, unitary invariant properties of the set of input spectral functions, as described in^18^. As the case with mixed states describing the internal degrees of freedom is more intricate^25^, here we will focus on the case of pure spectral functions.

In what follows, we describe in more detail the case of *n* = 3 photons, as it is the simplest case where multiphoton effects play a role. As discussed in^18^, the indistinguishability scenario for a 3-photon experiment is completely characterized by a Gram matrix:

1 $$\begin{array}{rcl}{G}^{{\prime} } & = & \left(\begin{array}{rcl}1 & | \langle {\psi }_{1}| {\psi }_{2}\rangle | & | \langle {\psi }_{1}| {\psi }_{3}\rangle | \\ | \langle {\psi }_{1}| {\psi }_{2}\rangle | & 1 & \langle {\psi }_{2}| {\psi }_{3}\rangle \\ | \langle {\psi }_{1}| {\psi }_{3}\rangle | & {\langle {\psi }_{2}| {\psi }_{3}\rangle }^{* } & 1\end{array}\right)\\ & = & \left(\begin{array}{rcl}1 & \sqrt{{\Delta }_{12}} & \sqrt{{\Delta }_{13}}\\ \sqrt{{\Delta }_{12}} & 1 & \sqrt{{\Delta }_{23}}{e}^{i\varphi }\\ \sqrt{{\Delta }_{13}} & \sqrt{{\Delta }_{23}}{e}^{-i\varphi } & 1\end{array}\right)\end{array}$$

parameterized in terms of the two-photon overlaps ${\Delta }_{ij}={| \langle {\psi }_{i}| {\psi }_{j}\rangle | }^{2}$ and the phase *φ* of the single nontrivial 3-photon Bargmann invariant

2 $${\Delta }_{123}=\langle {\psi }_{1}| {\psi }_{2}\rangle \langle {\psi }_{2}| {\psi }_{3}\rangle \langle {\psi }_{3}| {\psi }_{1}\rangle =\sqrt{{\Delta }_{12}{\Delta }_{13}{\Delta }_{23}}{e}^{i\varphi }$$

The phase of Δ_123_ is known as a collective photonic phase^14^, and is not accessible in experiments with fewer than 3 photons. We note that the phase *φ* cannot be chosen arbitrarily; it was recently shown that the value of ∣Δ_123_∣ imposes constraints on the allowed values for *φ*, stemming from the fact that a Gram matrix is positive-semidefinite^26^ (see also Supplementary Note 1).

Here, we will investigate some counter-intuitive behaviors of two different figures of merit describing multiphoton bunching:

- Probability of bunching *p*_B_ (or collisions), defined as the probability that at least one output mode has two or more photons.
- Probability of full bunching ${p}_{{\mathrm{FB}}}^{(i)}$ in the *i*th output mode – the probability that all photons come out of the interferometer in output mode *i*.

**Fig. 1:**
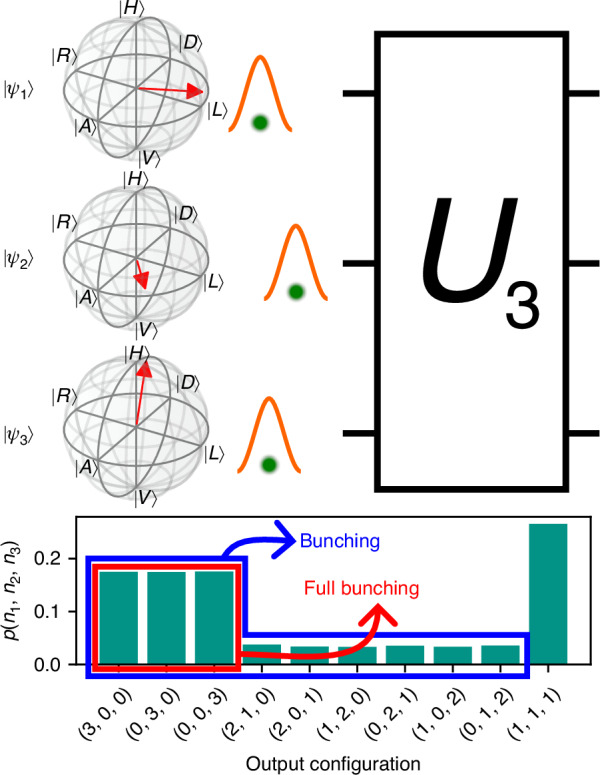
Characterization of the quantum interference of 3 photons evolving in a 3-mode interferometer. Three photons are injected in a linear optical network *U*_3_ implementing the 3-mode Fourier transform - i.e., a balanced tritter - and we sample from the distribution *p*(*n*_1_, *n*_2_, *n*_3_) of photon numbers at the output modes. From the measured photon number distribution of such a balanced interferometer, we extract the bunching probability *p*_B_ and the full bunching probability *p*_FB_, as shown in the lower panel. The polarization and time delays of the photons are manipulated to prepare indistinguishability scenarios described by a 3 × 3 Gram Matrix *G*

We will focus on analyzing these probabilities for the balanced tritter, i.e. a Fourier interferometer of three modes, with 1 photon per input mode (see Fig. 1). For a given indistinguishability scenario described by a Gram matrix of the form of Eq. (1), the bunching and full bunching probabilities depend only on the 3-photon Bargmann invariant and the average two-photon overlap

3 $$\overline{\Delta }=\frac{{\Delta }_{12}+{\Delta }_{23}+{\Delta }_{13}}{3}$$

and are given by^14^:

4 $${p}_{{\mathrm{B}}}=1+\frac{1}{9}[3\bar{\Delta }-4{\mathrm{cos}}(\varphi )| {\Delta }_{123}| -2]$$

5 $${p}_{{\mathrm{FB}}}^{(i)}=\frac{1}{27}[1+3\bar{\Delta }+2{\mathrm{cos}}(\varphi )| {\Delta }_{123}| ]$$

for any output mode *i* ∈ {1, 2, 3} (see Ref. ^14^). Note that for this balanced Fourier interferometer both *p*_B_ and *p*_FB_ depend on the overlaps Δ_*ij*_ only via the arithmetic mean $\overline{\Delta }$ and geometric mean ∣Δ_123_∣^2^.

#### Counter-intuitive behavior of *p*_B_

There are choices of interferometers for which partial indistinguishability scenarios have *higher* bunching probabilities *p*_B_ than the fully indistinguishable case. The most extreme case we found for 3-mode interferometers occurs for the balanced tritter, which is implemented experimentally in this work and has been previously used as a testbed for a first experimental analysis on this aspect^13^. In this case, *p*_B_ is actually *minimized* by perfectly indistinguishable photons. Hence, any increase in distinguishability (without necessarily requiring a non-trivial collective photonic phase) will increase the bunching probability. This is in sharp contrast with the expected behavior for Haar random matrices where, for example, distinguishable photons tend to bunch less than indistinguishable ones^12^. While counterexamples to this behavior can be found by generating random interferometers of small dimensions, this counter-intuitive behavior becomes rarer as the dimension grows (see Supplementary Note 2). It is also interesting to point out that for Fourier interferometers of even dimension with indistinguishable photons at the input, all allowed outcomes lead to bunching due to suppression laws^27,28^, meaning that full indistinguishability maximizes bunching in this case. Further examples of counter-intuitive bunching phenomena with a higher number of modes can be found for Fourier interferometers of certain odd dimensions, or real Hadamard interferometers with entries $\pm 1/\sqrt{n}$ which extremize the permanent^29^ (see Supplementary Note 2).

Going back to the three-mode Fourier interferometer, which is implemented experimentally in this work, it is natural to ask what kind of distinguishability scenario maximizes bunching. Using Eq. (4), it can be seen that the maximum probability of bunching is obtained for the largest possible negative value of the three-photon Bargmann invariant, a scenario characterized by equal overlaps Δ_*ij*_ = 1/4 and a collective photonic phase of *π*, which we explore experimentally in Section II B. We refer to Supplementary Note 3 for a detailed derivation of the maximum and minimum bunching probabilities for the three-mode Fourier interferometer.

#### Counter-intuitive behavior of *p*_FB_

It is known that full bunching probabilities ${p}_{FB}^{(i)}$ are always maximized for fully indistinguishable photons^15^, representing an intuitive behavior. However, *p*_FB_ fails to behave in an intuitive way in certain partial indistinguishability scenarios. This follows from a simple result due to Tichy^15^, relating full bunching probabilities in two different distinguishability scenarios described by Gram matrices *G*_1_, *G*_2_:

6 $$\frac{{p}_{{\mathrm{FB}}}^{(i)}({G}_{1})}{{p}_{{\mathrm{FB}}}^{(i)}({G}_{2})}=\frac{{\mathrm{Per}}({G}_{1})}{{\mathrm{Per}}({G}_{2})}$$

where Per( ⋅ ) denotes the matrix permanent function. The calculation of such permanents involves the product of the Gram matrix elements, thus leading in the 3-photon case to an expression for ${p}_{{\mathrm{FB}}}^{(i)}$ depending on the third-order Bargmann invariation as in Eq. (5). Additionally, Eq. (6) is a more general version of the full-bunching law discussed in^4^, that applies also to partial indistinguishability scenarios. The result above is independent of which interferometer is being used, and of the chosen output mode.

If we restrict ourselves to Gram matrices with real non-negative entries the intuitive behavior of full bunching is recovered: any decrease of two-photon overlaps Δ_*ij*_ = ∣〈*ψ*_*i*_∣*ψ*_*j*_〉∣^2^ results in diminished full bunching probabilities. However, the explicit dependency of Per(*G*) on the complex phases appearing in the Gram matrix, as can be seen from Eqs. (5), (6), clearly shows that pairwise distinguishability is not sufficient to understand full bunching phenomena. In fact, for three photons, if we fix the values of the overlaps Δ_*ij*_, a nontrivial collective phase *φ* *decreases* full bunching probabilities when compared to the same scenario with *φ* = 0. As a consequence of this, for any *φ* > 0, it is always possible to find scenarios where pairwise indistinguishability decreases, but at the same time, full bunching probabilities increase. More precisely, it follows from Eqs. (5), (6) that a sufficiently small decrease of pairwise indistinguishability, obtained by lowering the values of $\overline{\Delta }$ and ∣Δ_123_∣, can be compensated in the full bunching probability by tuning the collective phase *φ* towards 0. This effect is, of course, more prominent when we are allowed large variations of *φ*. For this reason, in the experimental sections, we focus on distinguishability scenarios targeting the preparation of states with Δ_*ij*_ = 1/4, which allows for arbitrary changes of the phase between [−*π*,*π*]^26^, to verify the theoretical predictions.

Interestingly, the condition *φ* ≠ 0, which we associate with counter-intuitive features of full bunching, is also associated with nonclassicality in other settings. As examples, the appearance of a non-trivial phase in a Bargmann invariant describing each scenario is required for anomalous weak values^19^, for nonclassical Kirkwood-Dirac quasiprobabilities^20^, and serves as a basis-independent witness of coherence^18^.

### Experimental verification of counterintuitive photon bunching effects

**Fig. 2:**
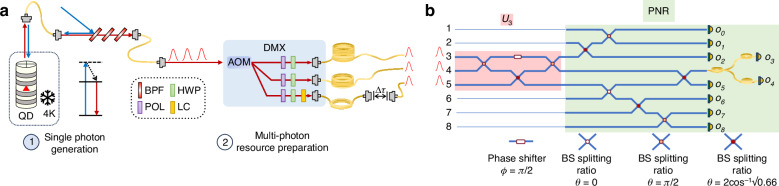
Experimental platform for the verification of counter-intuitive bunching properties: in this experimental verification, we employ the QOLOSSUS machine, consisting of several interconnected stages. **a** A Quantum Dot (QD) based single-photon source is excited in a non-resonant, longitudinal acoustic phonon assisted scheme, operating at cryogenic temperatures, and generating a stream of single photons at regular time intervals with a repetition rate of 79 MHz. Then, via a time-to-spatial demultiplexer (DMX) employing an acousto-optical modulator, the train of single photons is divided into three spatially separated modes, which are then temporally synchronized via properly tuned in-fiber delay loops. Inside the bulk geometry of the DMX setup, in order to independently control the spectral function $| {\psi }_{i}\rangle$ of each of the photons in our resource state, we add a polarizer and a half-wave plate for each channel (purple and green rectangles). Moreover, a liquid crystal (yellow rectangle) and a time-delay line are added to one of the channels to obtain full control of both time and polarization degrees of freedom. **b** The prepared states are then injected in an eight-mode, fully reconfigurable photonic integrated circuit. The circuit is programmed in two separate blocks. The red module is programmed as a three-mode balanced Fourier interferometer described by unitary ${\widetilde{U}}_{3}$ where interference occurs. Then each of its three output modes is manipulated separately in the green block, where, with an additional in-fiber BS, each output mode of the unitary ${\widetilde{U}}_{3}$ is split towards three independent threshold detectors in a balanced fashion. In such a way, we obtain a setup implementing a Pseudo Photon Number resolving (PPNR) measurement in order to reconstruct the full photon number output distribution *p*(*n*_1_, *n*_2_, *n*_3_). *Legend -* QD - Quantum Dot, BPF - Band Pass Filter, POL - Linear Polarizer, HWP - Half Wave Plate, LC - Liquid Crystal Retarder, AOM - Acousto-Optical Modulator

We now provide an experimental verification of counter-intuitive effects due to multiphoton quantum interference, which occur when more than two photons propagate within a multimode optical interferometer. The experiment has been carried out employing the QOLOSSUS photonic machine introduced in^30^, whose setup is shown in Fig. 2 and described in more detail in the Methods section and in Supplementary Note 4. Briefly, the experimental setup can be divided into three sequential stages, related to single-photon generation via a Quantum Dot (QD) based source^31–36^; multi-photon state preparation with a bulk time-to-spatial demultiplexing setup (DMX)^23,30,37,38^; and state evolution with pseudo-photon-number resolved detection implemented via a reconfigurable integrated photonic processor (IPP)^39–41^. Indeed, in order to engineer 3-photon states described by arbitrary Gram matrices, one can independently prepare each quantum state $| {\psi }_{i}\rangle$ using either polarization or time-delay. By introducing a set of half-wave plates, and an additional liquid crystal plus a time-delay on one of the photons (see Fig. 2a), one can prepare a set of three-photon states with the general form:

7 $$\begin{array}{rcl}| {\psi }_{1}\rangle & = & {| 0\rangle }_{t}\otimes (\cos (\alpha ){| 0\rangle }_{p}+\sin (\alpha ){| 1\rangle }_{p})\\ | {\psi }_{2}\rangle & = & {| 0\rangle }_{t}\otimes (\cos (\beta ){| 0\rangle }_{p}+\sin (\beta ){| 1\rangle }_{p})\\ | {\psi }_{3}\rangle & = & (x{| 0\rangle }_{t}+\sqrt{1-{x}^{2}}{| 1\rangle }_{t})\otimes \\ & \otimes & (\cos (\gamma ){| 0\rangle }_{p}+\sin (\gamma ){e}^{i\phi }{| 1\rangle }_{p})\end{array}$$

where $\{{| 0\rangle }_{p},{| 1\rangle }_{p}\}$ and $\{{| 0\rangle }_{t},{| 1\rangle }_{t}\}$ are orthogonal states in the polarization and time-delay basis, (*α*, *β*, *γ*, *ϕ*) are related to the orientation of the polarization states in the Bloch sphere, and *x* is associated to the relative time-delay. We note that a rigorous description of the time degree of freedom could be done considering a continuous temporal wave-function picture. However, operationally, our simplified notation equivalently identifies the fact that when photon 3 is time-delayed, its temporal wave function ${| {\mu }_{3}\rangle }_{t}$ will have $| {\langle {\mu }_{i}| {\mu }_{3}\rangle }_{t}{| }^{2}=x < 1$ with the other two photons (*i* = 1, 2). After preparing the input state, the photons are injected in a three-mode balanced Fourier interferometer implemented by suitably programming an 8-mode fully reconfigurable universal integrated chip^39–41^. The effective three-mode unitary ${\widetilde{U}}_{3}$ implemented on our device, reported in the Methods, has a trace fidelity of ${\mathcal{F}}=| Tr\{{U}_{3}^{\dagger }{\widetilde{U}}_{3}\}| /3=0.99922(4)$ with respect to the ideal transformation *U*_3_. By employing a pseudo-number resolved detection setup (see Fig. 2b), we are able to fully reconstruct the output probability distribution *p*(*n*_1_, *n*_2_, *n*_3_) and thereby recover both the total *full-bunching* probability *p*_FB_, associated with a given three-photon Gram-matrix *G*:

8 $${p}_{{\mathrm{FB}}}(G)=p(3,0,0;G)+p(0,3,0;G)+p(0,0,3;G)$$

and the *bunching* probability:

9 $${p}_{{\mathrm{B}}}(G)=1-p(1,1,1;G)$$

(see Fig. 1), where with *G* we indicated explicitly the dependence on the Gram matrix.

In what follows, we report results associated with different families of Gram matrices engineered by suitably preparing input states as in Eq. (7). We note that the overall quantum state prepared in our setup will depend on all internal degrees of freedom of the photon states. In other words, one can write the internal state of each photon *i* as $| {\psi }_{i}\rangle ={| {\chi }_{i}\rangle }_{o}\otimes {| {\mu }_{i}\rangle }_{t}\otimes {| {\nu }_{i}\rangle }_{p}$. Here, ${| \chi \rangle }_{o}$ is associated with the properties of the quantum dot emission itself and related to the maximum pairwise overlap achievable between photon pairs when they are aligned both in time and polarization, i.e., ${\Delta }_{ij}^{\max }=| \langle {\chi }_{i} {| {\chi }_{j}\rangle }_{o}{| }^{2}$. Conversely, ${| {\mu }_{i}\rangle }_{t}$ and ${| {\nu }_{i}\rangle }_{p}$ describe the internal states in the time and polarization degrees of freedom, respectively, which are tuned in the experiment. Hereafter, the pairwise overlaps ${\widetilde{\Delta }}_{ij}$ have been estimated by measuring the visibilities ${V}_{ij}^{HOM}$ of independent Hong-Ou-Mandel experiments carried out by suitably reconfiguring the IPP, and correcting for the bias effect introduced by the presence of a multi-photon component due to the non-zero second-order correlation function *g*^(2)^(0) ≠ 0. This effect is taken into account by using the approach of^42^, which evaluates the impact of this imperfection in the general scenario where the noise photon has an overlap *M*_*sn*_ with the principal photon emitted by the source. In the limit *M*_*sn*_ → 0, holding for the QD source employed in this experiment, this leads to the following relation between overlap, visibility, and second-order correlation function ${\widetilde{\Delta }}_{ij}=[{V}_{ij}^{{\mathrm{HOM}}}+{g}^{(2)}(0)]/[1-{g}^{(2)}(0)]$ with ${\widetilde{\Delta }}_{ij}\le 1$.

#### Experimental observation of counter-intuitive behavior of bunching

**Fig. 3:**
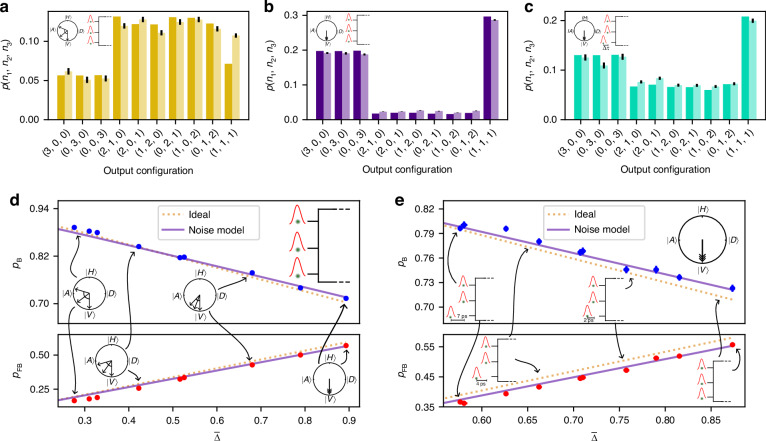
Measured and expected probabilities of bunching *p*_B_ and full bunching *p*_FB_, with control of photon polarization or time delay. The measured and simulated probability distributions *p*(*n*_1_, *n*_2_, *n*_3_), over the output Fock states, are shown: **a** in the case in which only the photon polarizations are manipulated by half-wave plates in order to obtain three states in the *x* − *z* plane of the Bloch sphere; **b** in the nearly indistinguishable case and **c**, in the case in which only an adjustable time delay is applied to one channel. The probabilities of bunching *p*_B_(*G*) = 1 − *p*(1, 1, 1; *G*) and full bunching *p*_FB_ = *p*(3, 0, 0; *G*) + *p*(0, 3, 0; *G*) + *p*(0, 0, 3; *G*) are obtained from the measured and simulated distributions, as a function of the average overlap $\overline{\Delta }$ which varies according to **d**, the polarization, and **e** the time delay configuration. Here, the (dashed) lines represent a (ideal) model of bunching and full-bunching expected at the output, with either a polarization-based or time-based Gram matrix preparation. As highlighted in the Supplementary Material, we can account for sources of experimental noise such as imperfections in the three-mode unitary implementation and a non-unit single photon purity of the source, with good agreement between experiment and the numerical prediction

The adoption of a polarization-insensitive technology for our IPP^41^ allows us to probe multi-photon interference effects generated by manipulation of the polarization degree of freedom in the state preparation stage. We generated triads of polarization states lying on a great circle of the Bloch sphere, e.g. the plane spanned by the eigenvectors of *σ*_*x*_ and *σ*_*z*_. In particular, using only the half-wave plates we can prepare states with *x* = 1, *γ* = 0 and *β* = *α* + δ in Eq. (7). Moreover, we choose *α* = δ leading to a real Bargmann invariant quantified as ${\Delta }_{123}\propto \cos (\alpha )\cos (\alpha )\cos (2\alpha )$, since with such a choice of states it can be shown that the triad phase associated with Δ_123_ is either 0 or *π*. The comparison between the measured and simulated probability distributions associated with two different Gram matrices with triad phase 0 or *π* are reported in Fig. 3a, b. In particular, they correspond to the nearly fully indistinguishable case, in which *α* = 0 and ${\Delta }_{ij}\approx {\Delta }_{ij}^{\max }$ (Fig. 3a), and to the case in which the pairwise overlaps are balanced, *α* = *π*/3 and ${\Delta }_{ij}\approx {\Delta }_{ij}^{\max }/4$ (Fig. 3b). In addition to these extremal conditions, we engineered ten more Gram Matrices by varying *α* in the interval [0, *π*/3]. Therefore, in Fig. 3d, we plot both the full bunching probability *p*_FB_(*G*) and the bunching probability *p*_B_(*G*) as a function of the average overlap $\overline{\Delta }$ associated to each experimental point, and we compare the trends with a theoretical prediction, which includes the main sources of imperfections according to a detailed model^23,30^ (see Supplementary Note 5 for further details). These measurements clearly show a counter-intuitive behavior of the bunching probability, since it *decreases* as we increase the average two-photon overlap.

A similar behavior can be obtained by only manipulating the temporal degree of freedom. In particular, by preparing each of the three streams of single photons with aligned $| V\rangle$ polarization and delaying one of them with respect to the other two, we can obtain the family of states in Eq. (7) corresponding to *α* = *β* = *γ* = 0, while *x* is tuned by the introduction of a fine temporal delay. As argued in^14^, the manipulation of the time degree of freedom does not affect the triad phase, which can only assume a null value with the use of a QD-based source (see Supplementary Note 4 for more details). Varying the parameter *x*, we engineered 10 different Gram matrices, and the results are reported in Fig. 3c–e. In particular, in Fig. 3c we compared the experimental and simulated probability distribution for the experimental data with the lowest average indistinguishability $\overline{\Delta }=0.576(3)$. In Fig. 3e we report both the bunching and full bunching probability as a function of $\overline{\Delta }$: again, one can see that the bunching probability counter-intuitively *decreases* as the average two-photon overlap increases.

#### Triad phase dependence of probabilities of bunching and full bunching

**Fig. 4:**
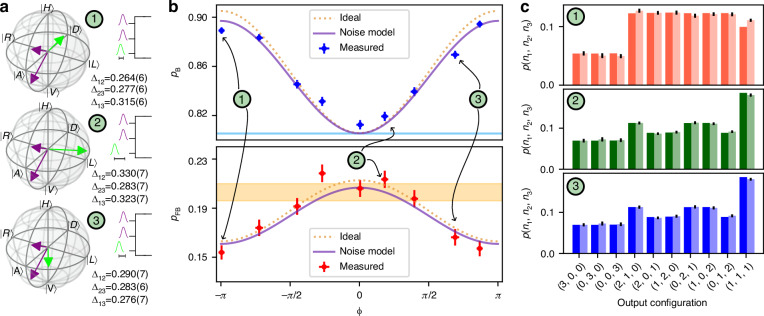
Role of the triad phase on the bunching *p*_B_ and full bunching *p*_FB_ probabilities. **a** Bloch sphere visualization of three polarization configurations obtained with half-wave plates and a liquid crystal, in order to tune the relative phase between the basis states and obtain complex-valued Gram matrices; via an additional time-delay, the pairwise photon overlaps Δ_*ij*_ are kept fixed in such a way that $\sqrt{{\Delta }_{ij}}\approx 1/2$. The measured overlaps are reported. **b** Probability of bunching *p*_B_ and full bunching *p*_FB_ as a function of the triad phase *φ*. In order to show non-trivial modulation of the (full) bunching probabilities due to a non-null triad phase, the blue and the orange regions represent the values attained by *p*_B_ and *p*_FB_, respectively, from numerical simulations which account for experimental imperfections and consider the experimentally measured overlaps Δ_*ij*_ while assuming *φ* = 0. Again, the (dashed) line represents the results of a numerical simulation of the experimentally tested Gram matrices showing a good agreement with the experimentally obtained distributions. **c** Measured and simulated probability distributions, respectively in full and opaque colors, over the possible output Fock states *p*(*n*_1_, *n*_2_, *n*_3_) for experimental Gram matrices characterized by $\sqrt{{\Delta }_{ij}}\approx \frac{1}{2}$ and an increasing triad phase *φ*, whose values are evaluated to be *φ* = { − 3.127, 0.578, 2.178}

As a next step, by acting simultaneously on both the time and polarization photonic degrees of freedom, we can highlight the effects due to a complex-valued Gram matrix. In particular, we focused on engineering a set of states (see Fig. 4a) with *α* = *π*/4 + δ, *β* = *π*/4 − δ and $\cos (\gamma )=\sin (\gamma )={2}^{-1/2}$ and a variable phase *ϕ* as in Eq. (7). This choice of parameters, for $\delta \approx \frac{\pi }{6}$, allows us to generate the family of Gram matrices characterized by $\sqrt{{\Delta }_{ij}}\approx \frac{1}{2}$ and a variable triad phase *φ*. This can be done by properly tuning the free parameters *x* and *ϕ*, where *x* affects only the overlaps while *ϕ* also affects the Gram matrix phase *φ*. We note that the latter parameter is in a one-to-one and monotonic relationship with the polarization state’s phase *ϕ*, and *ϕ* = *φ* holds only if *ϕ* = 0, ± *π*. In particular, we engineered 9 different state triplets, with the corresponding bunching and full bunching probabilities reported in Fig. 4b as a function of the measured triad phase. In Fig. 4c we show the comparison between the theoretical and experimental probability distribution associated with three different conditions characterized by $\sqrt{{\Delta }_{ij}}\approx \frac{1}{2}$ and the triad phases *φ* = { − 3.127, 0.578, 2.178}. As highlighted in Fig. 4b, by varying the triad phase *φ* carried by the Gram matrix, a counter-intuitive modulation of the (full) bunching probabilities is obtained, which cannot be directly related to the knowledge of only the overlaps Δ_*ij*_. Indeed, the experimentally obtained (full) bunching probability deviates consistently from the shaded regions, representing the expected bunching probabilities with a real-valued Gram matrix, while also being consistent with numerical simulations carried out considering a suitable noise model of our experimental implementation.

#### Experimental observation of counter-intuitive behavior of full bunching

In the previous sections, we have considered Gram matrices preparations describing three-photon states, as in Eq. (1), manipulated both in the polarization and time degrees of freedom. This has allowed us to directly verify a counter-intuitive behavior of the bunching probability *p*_*b*_, i.e. the probability that at least two out of three photons exit in the same output of a balanced Fourier interferometer, showing non-trivial trends as a function of the average overlap $\overline{\Delta }$ or the triad phase *φ* of the 3-photon Bargmann invariant as in Eq. (2). We now show that, by engineering appropriately both the overlaps and the phase of a 3-photon Gram matrix, it is possible to observe a counter-intuitive behavior arise even with respect to the *full-bunching* probability *p*_FB_. More specifically, we are able to find suitable preparations (A) and (B) such that ${p}_{FB}^{(A)}\ge {p}_{FB}^{(B)}$ even though ${\overline{\Delta }}^{(A)}\le {\overline{\Delta }}^{(B)}$ and $| {\Delta }_{123}^{(A)}| \le | {\Delta }_{123}^{(B)}|$. We start with the same state prepared in the previous section, i.e., with *α* = *π*/4 + δ, *β* = *π*/4 − δ and $\cos (\gamma )=\sin (\gamma )={2}^{-1/2}$.

**Fig. 5:**
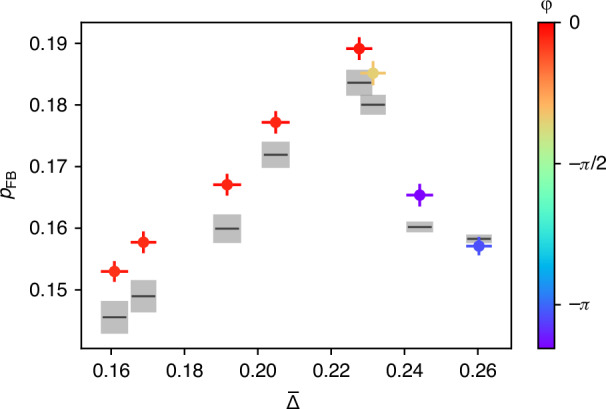
Counter-intuitive behavior of full bunching probabilities. Experimentally measured full-bunching probabilities *p*_FB_ at the output of a balanced Fourier interferometer (colored crosses), as a function of the measured average overlap $\overline{\Delta }$, showing that one can have decreasing probability of full bunching as the average overlap increases. The colormap shows the triad phase *φ* of the corresponding preparation. The measured results are compared to a numerical simulation that considers the experimentally measured Gram matrices

If one fixes $\delta =\frac{\pi }{6}$, for any choice of the polarization phase *ϕ* one can also tune the time-delay parameter *x* so that $\sqrt{{\Delta }_{ij}}\approx \frac{1}{2}$. By keeping fixed the average overlap ${\overline{\Delta }}_{(\phi ,x)}$, as in the previous section, one obtains a full bunching probability monotonically increasing with *ϕ*, when *ϕ* increases from − *π* to 0. Again, such a choice of *ϕ* is in a one-to-one correspondence with the induced triad phase *φ*. Interestingly, given a fixed value of *ϕ* and *x*, by increasing Δ up to the point in which the 3-state average overlap ${\overline{\Delta }}_{(\phi ,x)}(\delta )$ attains its minimum, *p*_FB_(*ϕ*, *x*, δ) behaves as a monotonically decreasing quantity with δ. Overall, this procedure gives us experimental access to Gram matrices whose *p*_FB_ decreases with increasing average overlap $\overline{\Delta }$. In Fig. 5, we report an experimental measurement of such behavior, where we show the experimentally measured *p*_FB_ as a function of the measured average overlap $\overline{\Delta }$. For the five left-most points in the Figure, which have been prepared with a fixed phase *ϕ* = *φ* ≈ 0, we find an intuitive monotonically increasing behavior of ${p}_{FB}(\overline{\Delta })$. However, by changing also the value of *φ* through the polarization phase *ϕ* one can observe a trend where the full-bunching probability decreases for increasing values of $\overline{\Delta }$ (the respective values of all relevant measured quantities and additional information can be found in Supplementary Note 6). In Supplementary Note 6 we show that *p*_FB_ shows the same qualitative counter-intuitive dependence as a function of the geometric mean ∣Δ_123_∣^2^ of the overlaps. Thus, our results show that by careful engineering of indistinguishability scenarios involving both polarization and time degrees of freedom, one can observe a counter-intuitive behavior of the full bunching probability.

## Discussion

In this work, we experimentally investigated counter-intuitive behavior of (full) bunching probabilities in 3-photon interference experiments with a balanced tritter. We observed that increasing distinguishability can lead to a larger probability of bunching events, where two or more photons coalesce in the same output mode. This can be associated with specific symmetries of the tritter, which forbid outcomes with two photons in the same output mode if the input photons are fully indistinguishable^28^. We have also experimentally verified the counter-intuitive behavior of full bunching, i.e., events with 3 photons in the same output, a more challenging experiment requiring a careful manipulation of pairwise overlaps between photonic wavefunctions describing polarization and time degrees of freedom. This enabled the demonstration of a scenario where an increase of pairwise indistinguishability together with a precise engineering of a triad phase^14^ actually resulted in a decrease of the full bunching probabilities. Our work highlights the complex interplay between bosonic indistinguishability and boson bunching phenomena, the understanding of which is crucial for the development of photonic quantum technologies such as quantum computing and quantum metrology due to the relevant roles of indistinguishability and relational information for photonic state generation and processing^18,26^.

## Materials and Methods

### Experimental apparatus

As stated in the main text, the experimental apparatus can be divided into three sequentially connected stages: generation of single-photon states, their manipulation with a time-to-spatial demultiplexing setup to generate a chosen 3-photon Gram matrix, state evolution, and reconstruction of the output probabilities obtained by suitably reconfiguring an integrated photonic interferometer.

### Single photon source

In this work single photons are generated via a Quantum Dot (QD) based source^31,32^. We employ an InGaAs QD^33–35^ operated at a repetition rate of *RR* ≈ 79 MHz in the so-called Longitudinal Acoustic (LA) phonon-assisted excitation scheme^36^. In this non-resonant excitation scheme, a pulsed pump laser is shone on the source with a wavelength of 927.2 nm, slightly blue-detuned from the relevant QD excitonic state, found to be at 927.8 nm. In such a way, filtering between the emitted single photons and the residual pump laser can be obtained via a set of three band-pass filters set at the single photon wavelength^36^. At the output of the single-photon source, we measured on avalanche photodiode detectors (APDs) a single-photon rate of ~ 3.5 MHz, achieving with a single-photon purity of *g*^(2)^(0) ~ 0.017(3) evaluated via a standard Hanbury-Brown-Twiss setup. The measured pairwise photon indistinguishability at source level was found to be *V*_HOM_ ~ 0.90(1), measured via a Hong-Ou-Mandel experiment implemented in a time-unbalanced Mach-Zehnder interferometer^2^ by interfering photons separated by $\Delta t=\frac{1}{RR}$.

### Three-photon resource preparation

Then, the single photon stream is directed to a time-to-spatial demultiplexing setup with a single-mode fiber. In this stage, photons are initially steered, by means of an acousto-optical-modulator, into one output channel at a time for a time duration of *T* ~ 180 ns, resulting in a total of three different occupied spatial modes. Thereafter, fiber delays are employed for the synchronization of the single photons on the different output channels of the device, akin to the scheme described in^23,30,37,38^. Note that in order to accomplish the task of this experiment, a polarization and a time delay control of each photon is implemented in this stage. The average pairwise photon indistinguishability at the output of the demultiplexing setup, due to large time separation, it is found to be *V* ~ 0.85.

### Gram matrix preparation

Indeed, for any Gram matrix of dimension *n* it is always possible to find a set of *n* quantum states $\{| {\psi }_{j}\rangle \}$, such that ${G}_{ij}=\langle {\psi }_{i} | {\psi }_{j}\rangle$. One way to obtain such *quantum realization* of a Gram matrix is via the so-called Cholesky decomposition^43^. Namely, the rank *r* of a Gram matrix corresponds to the dimension of the vector space spanned by the states $\{| {\psi }_{j}\rangle \}$, and there exist a unique decomposition

10 $$G=L{L}^{\dagger }$$

where *L* is a lower-triangular matrix. From the rows of *L*, we can read out the entries of the quantum states realizing the desired Gram matrix. For *n* = 3, the fact that *L* is lower triangular means that these have the form:

11 $$\begin{array}{rcl}| {\psi }_{1}\rangle & = & | 1\rangle \\ | {\psi }_{2}\rangle & = & {\alpha }_{1}| 1\rangle +{\alpha }_{2}| 2\rangle \\ | {\psi }_{3}\rangle & = & {\beta }_{1}| 1\rangle +{\beta }_{2}| 2\rangle +{\beta }_{3}| 3\rangle \end{array}$$

if the matrix has full rank. If it has rank *r* = 2, then *β*_3_ = 0. As said, a representation of this form (resulting in a lower-triangular *L*) is unique. However, if we apply the same unitary *U* to all the states, we obtain a different physical realization of the same Gram matrix.

To physically realize the three-photon states necessary to generate any given *n* = 3 Gram matrix, we employed polarization and time delay control to achieve tuning of the state $\{| {\psi }_{j}\rangle \}$ of each component comprising our three-photon resource. Specifically, polarization control consists of a linear polarizer and a half-wave plate (HWP), together with a liquid crystal (LC) retarder placed in the third output of the DMX. Here, an additional tunable delay line is inserted to reduce, if needed, the degree of distinguishability in the time degree of freedom. In such a way, we can prepare states with the general form of Eq. (7). Physically, tuning of the polarization parameters {*α*, *β*, *γ*} is obtained via the HWPs; tuning of *ϕ* is induced by the LC and finally, the parameter *x* can be modulated via the time-delay-line.

### State evolution and measurement

Then, the three photon resource with each photon prepared in state $\{| {\psi }_{j}\rangle \}$ is injected in modes {3, 4, 5} of an eight-mode fully reconfigurable integrated photonic processor (IPP)^30^, obtained via femto-second laser writing^44^. The IPP is composed by a mesh of 28 Mach-Zehnder interferometers, arranged in a rectangular shape which has been shown^39^ to be able to implement any unitary transformation between input and output modes. Our IPP is built to operate in a polarization-insensitive fashion^41^ meaning that the unitary transformation implemented by the circuit does not change whatever polarization state is injected at the input. This unique property of femtosecond-laser-written photonic circuits decouples the polarization and the path degrees of freedom of the photons, and thus one can use the former to prepare the desired Gram matrix without affecting the tritter and pseudo-number-resolving transformations implemented on chip. Programmability of the circuit with high-fidelity to any given target unitary *U*_8_ is obtained via thermo-optical phase shifters^40^ whose induced optical response has been characterized in order to be able to implement any given transformation. The experiment involved programming the device in two different blocks, as described in the main text.

We characterized the effective unitary ${\widetilde{U}}_{3}$ being programmed into the device with standard methods^45^ relying on the measurement of the pairwise output visibility when injecting a two photon state in the input of the interferometer and adapted to our setup as already described in^30^. We found the moduli of the effective unitary to be:

12 $$| {\widetilde{U}}_{3}| =\left(\begin{array}{lll}0.5998(9) & 0.5563(9) & 0.575(1)\\ 0.5471(8) & 0.5906(9) & 0.593(1)\\ 0.584(1) & 0.585(1) & 0.563(1)\end{array}\right)$$

with phases

13 $$\arg ({\widetilde{U}}_{3})=\left(\begin{array}{rll}0 & 0 & 0\\ 0 & 2.135(4) & -2.106(4)\\ 0 & -2.081(4) & 2.158(4)\end{array}\right)$$

achieving a fidelity with the ideal balanced three-mode Fourier interferometer:

14 $${U}_{3}=\frac{1}{\sqrt{3}}\left(\begin{array}{rll}1 & 1 & 1\\ 1 & {e}^{i2\pi /3} & {e}^{i4\pi /3}\\ 1 & {e}^{i4\pi /3} & {e}^{i8\pi /3}\end{array}\right)$$

of $${\mathcal{F}}=| Tr\{{U}_{3}^{\dagger }{\widetilde{U}}_{3}\}| /3=0.99922(4)$$.

### Reconstruction of the photon number distribution

In our implementation, we employ a Pseudo Photon number resolving (PPNR) technique, following the same approach of Ref. ^30^. The goal is to employ threshold detectors to be able to reconstruct the photon number distribution *p*(*n*_1_, *n*_2_, *n*_3_), with ∑_*i*_*n*_*i*_ = 3, at the output of the implemented three-mode unitary transformation ${\widetilde{U}}_{3}$. To this end, each output mode of ${\widetilde{U}}_{3}$ is independently split - through a beam splitter cascade providing balanced splitting ratios - towards a set of three auxiliary modes. The output of each auxiliary mode is then measured via avalanche photodiodes (APDs), i.e. threshold detectors, with a quantum efficiency typically ranging in the interval *η* ~ 0.3 − 0.35 for the employed photon wavelength. In such a way, the presence of 1, 2, or 3 photons in one of the output modes of ${\widetilde{U}}_{3}$ can be probabilistically discriminated by counting the number of 1-, 2- and 3-fold events between the detectors placed in the corresponding auxiliary modes. Note that the whole experiment is run in a regime where only three-fold events - registered between three of a total of nine detectors - are considered. Overall, one can solve a combinatorial problem to find the probability that a given photon-number configuration (*n*_1_, *n*_2_, *n*_3_) is detected by three independent APDs in the PPNR configuration. Namely, the probability *P*^click^({*n*_1_, *n*_2_, *n*_3_}) of detecting as a three-fold event a given output configuration {*n*_1_, *n*_2_, *n*_3_} - or its permutations - can be written as:

15 $$\left\{\begin{array}{l}{P}^{\mathrm{click}}(\{3,0,0\})=2{\eta }^{3}/9\\ {P}^{\mathrm{click}}(\{2,1,0\})=2{\eta }^{3}/3\\ {P}^{\mathrm{click}}(\{1,1,1\})={\eta }^{3}\end{array}\right.$$

Therefore, in order to reconstruct the full photon-number distribution via PPNR, the number of registered three-fold events $\widetilde{N}({n}_{1},{n}_{2},{n}_{3})$ have to be corrected as:

16 $$N({n}_{1},{n}_{2},{n}_{3})=\widetilde{N}({n}_{1},{n}_{2},{n}_{3})/{P}^{click}(\{{n}_{1},{n}_{2},{n}_{3}\})$$

Such a three-fold event table is then normalized to obtain an experimental estimation of the relevant photon number distribution *p*(*n*_1_, *n*_2_, *n*_3_).

## Supplementary information

Supplementary Information: Experimental observation of counter-intuitive features of photonic bunching

## Data Availability

The data that support the findings of this study are available from the corresponding author upon reasonable request.
