# Supplementary material for: Experimental observation of counter-intuitive features of photonic bunching
Source: Light Sci Appl. 2026 Jun 29;15:292. doi: 10.1038/s41377-026-02250-4 (PMC13315700; doi:10.1038/s41377-026-02250-4)
Supplement: Supplementary file 1 — Supplementary Information: Experimental observation of counter-intuitive features of photonic bunching [file 41377_2026_2250_MOESM1_ESM.pdf]

Supplementary Information: Experimental observation of counter-intuitive features of photonic bunching

Giovanni Rodari,[1] Carlos Fernandes,[2] Eugenio Caruccio,[1] Alessia Suprano,[1] Francesco Hoch,[1] Taira Giordani,[1] Gonzalo Carvacho,[1] Riccardo Albiero,[3] Niki Di Giano,[3,4] Giacomo Corrielli,[3] Francesco Ceccarelli,[3] Roberto Osellame,[3] Daniel J. Brod,[5] Leonardo Novo,[2] Nicolò Spagnolo,[1,*] Ernesto F. Galvão,[2,5,†] and Fabio Sciarrino[1]

[1] *Dipartimento di Fisica, Sapienza Università di Roma, Piazzale Aldo Moro 5, I-00185 Roma, Italy*
[2] *International Iberian Nanotechnology Laboratory (INL) Av. Mestre José Veiga s/n, 4715-330 Braga, Portugal*
[3] *Istituto di Fotonica e Nanotecnologie, Consiglio Nazionale delle Ricerche (IFN-CNR), Piazza Leonardo da Vinci 32, I-20133 Milano, Italy*
[4] *Dipartimento di Fisica, Politecnico di Milano, Piazza Leonardo da Vinci 32, 20133 Milano, Italy*
[5] *Instituto de Física, Universidade Federal Fluminense, Niterói – RJ, Brazil*

Supplementary Note 1. General Remarks about the Gram Matrices

In what follows, we provide some general remarks about the space of reachable Gram matrices in a 3-photon setup. These are some general properties about Gram matrices [1]:

- A 3x3 Gram matrix G is described by the inner products amongst a set of three vectors, i.e. quantum states $|\psi_{1,2,3}\rangle$. A Gram matrix is then invariant upon applying a unitary transformation to its generating vectors.
- A Gram matrix, generated by vector defined on a complex field, is a Hermitian and positive semi-definite (PSD) matrix.

The third condition becomes interesting when trying to find a physical realization of a generic Gram matrix G. By being Hermitian, a Gram matrix must have real eigenvalues $\lambda_i \in \mathcal{R}$; since it is also a PSD matrix, such eigenvalues must be also positive $\lambda_i \geq 0$. Since the determinant of a matrix can be written as the product of its eigenvalues, if we write:

$$G' = \begin{pmatrix} 1 & x & y \\ x & 1 & ze^{i\varphi} \\ y & ze^{-i\varphi} & 1 \end{pmatrix} \tag{1}$$

a necessary condition for its physical realizability is that:

$$\det(G') = 1 + 2xyz\cos(\varphi) - x^2 - y^2 - z^2 \geq 0 \tag{2}$$

which in turn gives an algebraic inequality for the feasible values of the triad phase φ:

$$\cos(\varphi) \geq \frac{x^2 + y^2 + z^2 - 1}{2xyz} \tag{3}$$

The equality in the above condition is what traces the droplet-shaped boundary in Fig. S1, as also discussed in more detail in [2]. Restricting ourselves to a set of "balanced" Gram matrices, i.e., such that $x = y = z$, this means that:

$$\cos(\varphi) \geq \frac{3x^2 - 1}{2x^3} \tag{4}$$

which can be always fulfilled iff $0 < x \leq 0.5$.

* nicolo.spagnolo@uniroma1.it
† ernestogalvao@id.uff.br

Supplementary Note 2. Bunching probabilities in random and balanced interferometers

It is natural to ask how easy it is to find interferometers where bunching probability is not maximized for fully indistinguishable photons. In this Section we consider this question for larger system sizes than the one implemented experimentally, where $n = 3$. We restrict ourselves to the case where the input to the interferometer consists of a single photon per mode (and thus the numbers of photons and of modes are equal), and leave it as an interesting open question a full investigation when some modes can be initialized with no photons. We also focus on the specific case of comparing bunching probabilities between fully indistinguishable and fully distinguishable bosons.

We generated 100001 Haar-random unitary matrices of size $n \times n$ for each n from 3 to 9 and counted how many demonstrate counter-intuitive behaviour, i.e. when the probability of bunching is higher for distinguishable particles than for indistinguishable ones.

The number of matrices of each dimension resulting in counter-intuitive behaviour are reported in Table S1. For matrices of dimension 6 or greater no matrices displaying counter intuitive behaviour were generated, suggesting such matrices become vanishingly rare even for systems of modest size.

dimension	3	4	5	≥ 6
# of matrices	1890	217	17	0

Table S1. **Number of matrices resulting in counter-intuitive behaviour.** Results of a numerical simulation showing the number of matrices showing counter-intuitive features, from a sample of 100001 Haar-random unitaries, as a function of the number of modes m (dimension).

In spite of the rarity of counter-intuitive behaviour outside of small systems, there are certain families of matrices which display counter-intuitive behaviour even for larger dimension. An example of such matrices are Fourier matrices F_n for certain odd dimensions n (for even dimensions the permanent vanishes and the bunching probability for indistinguishable photons is 1). The values of the bunching probabilities for distinguishable and indistinguishable photons are given by $p_{\mathrm{B}}^{(\mathrm{dist.})} = 1 - n!/n^n$ and $p_{\mathrm{B}}^{(\mathrm{ind.})} = 1 - |\mathrm{Per}(F_n)|^2$, respectively. As these values tend both to 1 asymptotically, in Table S2 we present the values of the *antibunching probabilities*, that is the probability of event (11...1) comparing the cases of distinguishable vs indistinguishable photons. We can find counter-intuitive behavior for dimensions 3, 7, 11, 13, 17 and 21.

n	Distinguishable	Indistinguishable	Gap	Indist./Dist. Ratio
3	0.2222	0.333	-0.1111	1.5
5	3.80e-2	8.000e-3	3.040e-2	0.2083
7	6.120e-3	1.339e-2	-7.267e-3	2.188
9	9.367e-4	1.694e-5	9.197e-4	0.01808
11	1.399e-4	1.604e-4	-2.050e-5	1.147
13	2.056e-5	1.020e-4	-8.142e-5	4.960
15	2.986e-6	2.107e-9	2.984e-6	7.057e-4
17	4.300e-7	7.689e-7	-3.389e-7	1.788
19	6.149e-8	1.032e-8	5.116e-8	0.1679
21	8.745e-9	3.54e-9	5.204e-9	0.4049

Table S2. **Probability of antibunching events, i.e. outcome $(11\ldots1)$, in Fourier interferometers with one photon per input mode, in the case of distinguishable and indistinguishable photons.** The difference between the values and their ratio is also presented. A negative gap value (or a ratio larger than 1), means that $p^{(\mathrm{dist.})}(11\ldots1) < p^{(\mathrm{ind.})}(11\ldots1)$ showing examples of counter-intuitive behavior where distinguishable particles lead to a higher bunching probability than indistinguishable bosons.

Exploiting existing work on permanents of real-valued Hadamard matrices, it is also possible to compute bunching probabilities for Hadamard interferometers, a balanced n-mode interferometer with entries $\pm 1/\sqrt{n}$. These matrices only exist if $n \leq 2$ or n divides 4. For a given dimension there may exist several different equivalence classes of Hadamard matrices with different values of the permanent. These values have been computed explicitly in [3] for all real-valued Hadamard matrices up to dimension 28. By taking the equivalence class with the largest value for the permanent in a given dimension, it is possible to find examples where $p_{\mathrm{B}}^{(\mathrm{dist.})} > p_{\mathrm{B}}^{(\mathrm{ind.})}$ for dimensions 4, 8, 12, 16, 20, 24 and 28 (see Table S3).

n	Distinguishable	Indistinguishable	Gap	Indist./Dist. Ratio
2	0.5000	0	0.500	0
4	9.375e-2	0.2500	-0.1563	2.667
8	2.403e-3	8.789e-3	-6.386e-3	3.656
12	5.372e-5	2.3814e-4	-1.844e-4	4.433
16	1.134e-6	1.393e-4	-1.382e-4	122.8
20	2.320e-8	4.591e-10	2.274e-8	1.979e-2
24	4.652e-10	1.461e-8	-1.412e-8	31.40
28	1.34877e-11	9.19848e-12	-4.28922e-12	1.4663

Table S3. **Probability of antibunching events, i.e. outcome (11...1), in selected examples of real-valued Hadamard interferometers with one photon per input mode, for the cases of perfectly distinguishable and perfectly indistinguishable photons.** For each dimension we choose the real-valued Hadamard interferometer with the largest permanent using the values listed in [3]. The difference between the probabilities and their ratio is also presented. A negative gap value (or a ratio larger than 1), means that $p^{(\mathrm{dist.})}(11\ldots1) < p^{(\mathrm{ind.})}(11\ldots1)$ showing examples of counter-intuitive behavior where distinguishable particles lead to a higher bunching probability than indistinguishable bosons.

Finally, we point out that though our numerical investigation only revealed this counter-intuitive behaviour up to small matrix dimensions, it can be shown to happen for arbitrarily large system sizes. A trivial construction that achieves this is a $3n$-mode interferometer consisting of n parallel tritters, though the corresponding gap is exponentially supressed. If for each tritter the probability of observing outcome (111) is 0.2222 (resp. 0.3333) in the distinguishable (resp. indistinguishable) case, then the probability of this happening coincidentally for all n tritters is 0.2222^n (resp. 0.3333^n), with a gap of $0.2222^n - 0.3333^n$.

Supplementary Note 3. Maximum and minimum of the bunching probability

In this section, for ease of notation we will denote the Bargmann invariant as $\Delta_{123} = re^{i\varphi}$. The theorem stating that the arithmetic mean must be greater or equal to the geometric mean gives us the relation

$$r^{\frac{2}{3}} \leq \bar{\Delta} \tag{5}$$

In turn, the requirement that the Gram matrix be positive definite implies its determinant must be positive and therefore

$$\bar{\Delta} \leq \frac{1 + 2r\cos\varphi}{3} \tag{6}$$

The last two results provide strict upper and lower bounds for the average overlap $\bar{\Delta}$ for a given absolute value of the Bargmann invariant r:

$$r^{\frac{2}{3}} \leq \bar{\Delta} \leq \frac{1 + 2r\cos\varphi}{3} \tag{7}$$

Replacing in the expression for the bunching probability we have

$$\frac{7 - 4r\cos\varphi + 3r^{\frac{2}{3}}}{9} \leq p_{\mathrm{B}} \leq \frac{8 - 2r\cos\varphi}{9} \tag{8}$$

We can find the greatest bunching probability by maximizing the upper bound. This can be done by minimizing the real part of the Bargmann invariant, which is achieved for $r = 1/8$ and $\varphi = \pi$ [2], corresponding to a bunching probability $p_{\mathrm{B}} = 11/12$. The minimum of the bunching probability is analogously achieved by minimizing the lower bound. For any value of r, we can decrease the lower bound by setting $\varphi = 0$. Hence the minimum is obtained as

$$P_{\min} = \min_{0 \leq r \leq 1} \left(\frac{7 - 4r + 3r^{\frac{2}{3}}}{9} \right) \tag{9}$$

This is minimized for $r = 1$, corresponding to fully indistinguishable photons, and a minimum bunching probability of $p_{\mathrm{B}} = 2/3$.

Supplementary Note 4. Experimental setup

As stated in the main text, in order to engineer a Gram matrix different from the one where all entries are equal to 1, i.e. the perfectly indistinguishable case, one must be able to control the input photons' spectral functions $|\psi_i\rangle$, which are then injected into an optical interferometer in which the photons interfere. Then, one must also be able to reconstruct the full probability distribution - in the photon number space - at the output of the interferometer. The experimental setup can be divided into three sequentially connected stages, related to single-photon generation via a Quantum Dot-based source; multi-photon state preparation with a bulk time-to-spatial demultiplexing setup; and state evolution with pseudo-photon-number resolved detection implemented via an integrated reconfigurable interferometric mesh.

A stream of single photons is generated via an InGaAs Quantum Dot (QD) [4–8] operated at a repetition rate of ≈ 79 MHz. More specifically, we employ the so-called Longitudinal Acoustic (LA) phonon-assisted excitation scheme [9]: a pulsed pump laser is shone on the source with a wavelength of 927.2 nm, slightly blue-detuned from the relevant QD excitonic state - found to be at 927.8 nm. In such a way, filtering between the emitted single photons and the residual pump laser can be obtained via a set of three band-pass filters set at the single photon wavelength. At the output of the source, we measured on avalanche photodiode detectors a single-photon rate of ≈ 3.5 MHz, achieving with a single photon purity of $g^{(2)}(0) \approx 0.02$ evaluated via a standard Hanbury-Brown-Twiss setup together with a pairwise photon indistinguishability of $V_{\mathrm{HOM}} \approx 0.90$ measured via a Hong-Ou-Mandel experiment implemented in a time-unbalanced Mach-Zehnder interferometer [10].

Then, the single photons are directed to a time-to-spatial demultiplexing setup [11–14] via a single-mode fiber. In this stage, the photons are initially steered, by means of an acousto-optical-modulator, into one output channel at a time for a time duration of $T \sim 180$ ns, resulting in a total of three different occupied spatial modes. Thereafter, fiber delays are employed for the synchronization of the single photons on the different output channels of the device. Moreover, a polarization and a time delay control are implemented in this stage in order to accomplish the task of this experiment. Specifically, the polarization control consists of a polarizer and a waveplate $\lambda/2$ (respectively, purple and green rectangles in Fig. 2 of the main text) on each channel, a liquid crystal (yellow rectangle in Fig. 2 of the main text) on the same channel of the subsequently time delay line.

At this point, the three trains of single photons, each associated with a certain spectral function, are injected into an eight-mode fully reconfigurable integrated photonic processor (IPP) with rectangular shape [15]. The circuit is fabricated by means of the femtosecond laser writing (FLW) technique [16] and it is composed of a rectangular mesh of 28 Mach-Zehnder interferometer. Each unit cell consists of two directional couplers, acting as 50:50 beam splitters, and two phase shifters. The phase shifter can be thermally controlled [16], allowing the possibility to reconfigure fully the unit cell and, therefore, the behaviour of the entire circuit. The red area in Fig. 2 of the main text is dedicated to the implementation of the three mode balanced Fourier unitary matrix, while the green is involved in Pseudo-Number Resolving detection. Finally, the outgoing photons are detected by avalanche photodiode (APD).

Supplementary Note 5. Modeling the experiment

Here we discuss a model employed to retrieve the predicted values of the experimentally measured quantities throughout the manuscript, taking into account the main experimental imperfections of the apparatus. The ideal scenario for the experiment corresponds to the implementation of the three-mode Fourier transformation U_3, with elements $(U_3)_{j,k} = e^{\imath 2\pi jk/3}/\sqrt{3}$, while measuring the output events for a three-photon input state given by a specific Gram matrix preparation. In the employed apparatus, one needs to consider the presence of some noise sources which modifies the output measurements from the ideal scenario described above (see also [12]).

Non-ideal implementation of the Fourier interferometer. In our implementation, the Fourier interferometer is realized by programming the 8-mode universal processor to realize such a transformation. This leads to an effective implemented unitary matrix $\tilde{U}_3$. The effective implemented unitary $\tilde{U}_3$ is taken into account in the experimental model as the actual transfer matrix between input and output modes.

Multiphoton contributions from the source. The second source of noise is related to the multiphoton contributions from the quantum dot source. More specifically, a second photon can be present in each time-bin with a small probability $p^{(2)}$, thus leading to the need of appropriate corrections in the output probabilities. For quantum dot sources [17], this noise photon is found to be distinguishable from the main photons. The ratio between the probability of emitting a single photon ($p^{(1)}$) and the one of finding also a noise photon ($p^{(2)}$) can be retrieved from the second order correlation parameter $g^{(2)}(0)$ as $g^{(2)}(0) = 2p^{(1)}/(p^{(1)} + 2p^{(2)})^2$. In the complete model for the experiment, we have then taken into account terms due to multiphoton emission by considering all possible contributions to the output probability distributions.

Losses. In our experiment, losses between the different arms are found to be almost balanced. One can thus consider the approximation of balanced losses for our apparatus, and exploit the result of [18]. Losses in the model can be thus made to commute with the interferometer and the demultiplexing module, and placed as a unique loss parameter at the output of the source. As a relevant note, the three-photon experiment is performed in a post-selected scenario, when three photons are measured at the output. Given that multiphoton contributions are present in the source, the role of losses is not limited to a reduction of the detected signal of a factor η^n, being η the overall transmission per photon. Indeed, losses also have the effect of changing the relative weights of the different terms due to the presence of multiphoton contributions. The only unbalanced losses contributions are due to detection efficiency associated to the probabilistic photon counting apparatus, and this is taken into account by directly correcting the experimental measured probabilities.

Estimating the system parameters. Within the experiment, the parameters to evaluate the predicted results from the model are retrieved as follows. The probabilities $p^{(1)}$ and $p^{(2)}$ are retrieved from the source brightness $B \sim p^{(1)} + p^{(2)}$ and the second order correlation parameter $g^{(2)}(0) = 2p^{(1)}/(p^{(1)} + 2p^{(2)})^2$, estimated from a Hanbury-Brown-Twiss experiment on the quantum dot source. Typical values for the $g^{(2)}(0)$ parameter are found in the range $g^{(2)}(0) \in (0.015, 0.025)$. Furthermore, losses are directed estimated via a loss budget of the apparatus, leading to an overall efficiency of around 0.011. We have that the fibered brightness of the source is $\eta_{\mathrm{F}} \approx 0.13$; a transmission efficiency of the DMX setup of $\eta_{\mathrm{DMX}} \approx 0.8$; an efficiency of the polarization filtering of $\eta_{\mathrm{pol}} \approx 0.7$; an integrated chip transmission of $\eta_{\mathrm{chip}} \approx 0.5$ and a detection efficiency of $\eta_{\mathrm{det}} \approx 0.35$. With an additional in-fiber loss of $\eta_{\mathrm{I}} \approx 0.85$ due to splicing and mating sleeves, we have overall: $\eta = \eta_{\mathrm{F}}\eta_{\mathrm{DMX}}\eta_{\mathrm{pol}}\eta_{\mathrm{I}}\eta_{\mathrm{chip}}\eta_{\mathrm{det}} \approx 0.011$.

The effective transformation $\tilde{U}_3$ implemented via the reconfigurable processor has been reconstructed via tomographic techniques, where the obtained matrix elements are reported in the Methods. The fidelity between the Fourier transformation U_3 and the implemented one $\tilde{U}_3$ is found to be $F = |\,\mathrm{Tr}\{U_3^{\dagger}\tilde{U}_3\}|/3 = 0.99922(4)$, thus showing that the realized evolution is very close to the ideal one leading to very minor corrections in the output probabilities.

Finally, one needs to estimate the effective parameters of the Gram matrix, that is, the two-photon overlaps Δ_{ij} and the complex phase φ. The two photon overlaps are estimated by measuring the pair-wise Hong-Ou-Mandel visibilities between the photons at the output of the demultiplexer. A second approach has been also used, based on a simple postprocessing of the probabilities of the different outcomes at the output of the interferometer, which allows to estimate both real and imaginary parts of the third-order Bargmann invariant Δ_{123}. More specifically, for a given Gram matrix G as parametrized in the main text, let $p(n_1, n_2, n_3)$ denote the probability of observing outcome (n_1, n_2, n_3) at the output of the Fourier interferometer. Using the explicit expression of the outcome probabilities from [19] we can define the following probabilities:

$$P_{\mathrm{A}} = p(1,1,1) + p(3,0,0) + p(0,3,0) + p(0,0,3) = \frac{1}{3} + \frac{2}{3}|\Delta_{123}|\cos(\varphi) \tag{10}$$

$$P_{\mathrm{B}} = p(0,2,1) + p(2,1,0) + p(1,0,2) = \frac{1}{3} - \frac{2}{3}|\Delta_{123}|\cos(\varphi + \pi/3) \tag{11}$$

$$P_{\mathrm{C}} = p(1,2,0) + p(0,1,2) + p(2,0,1) = \frac{1}{3} - \frac{2}{3}|\Delta_{123}|\cos(\varphi - \pi/3) \tag{12}$$

The Bargmann invariant can be extracted from these values via the following relation:

$$\Delta_{123}^{N} = |\Delta_{123}^{N}|e^{i\varphi} = P_{\mathrm{A}} + P_{\mathrm{B}}e^{i2\pi/3} + P_{\mathrm{C}}e^{i4\pi/3} \tag{13}$$

From here, it follows that the triad phase associated to a given indistinguishability scenario obtained via modulation of both the polarization and the time degree of freedom of the photonic resources, thus generally associated with a complex-valued Gram matrix S, can be estimated experimentally as $\varphi = \arg \Delta_{123}^{N}$.

This set of estimated parameters have been then used to perform the predictions from the model, reported in Figs. 3-5 of the main text. In particular, the two independent estimation of the overlaps and third-order Bargmann invariant have been combined simultaneously to define the indistinguishability properties of the input three-photon states.

Supplementary Note 6. Experimental data

The results regarding the counter-intuitive behavior of photonic bunching were presented in the main text. In this section, we present the measured data used in the figures of the main text. In particular, Tabs. S4 and S5 contain the data of bunching and full bunching probabilities, p_{B} and p_{FB}, as functions of the mean overlap $\bar{\Delta}$, when operating only on the polarization and time degrees of freedom, respectively. Next, the modulation of bunching and full bunching probabilities, p_{B} and p_{FB}, as the triad phase φ varies while keeping the average overlap $\bar{\Delta}$ approximately fixed, is

Δ_{12}	Δ_{23}	Δ_{31}	$\bar{\Delta}$	$\lvert\Delta_{123}^{V}\rvert$	$\lvert\Delta_{123}^{N}\rvert$	φ	p_B	p_{FB}
0.269(4)	0.316(4)	0.243 (4)	0.276(3)	0.144(2)	0.092(9)	2.98 (8)	0.893(3)	0.165(6)
0.461(4)	0.340(4)	0.129 (4)	0.310(3)	0.142(3)	0.063(4)	-2.71 (6)	0.883(1)	0.179(3)
0.476(4)	0.422(4)	0.086 (4)	0.328(3)	0.132(4)	0.046(4)	-2.51 (8)	0.880(1)	0.188(3)
0.563(4)	0.570(4)	0.135 (4)	0.423(3)	0.208(4)	0.120(5)	0.126 (3)	0.844(2)	0.257(4)
0.658(4)	0.670(4)	0.220 (5)	0.516(3)	0.312(4)	0.264(5)	0.161 (1)	0.816(2)	0.323(4)
0.595(3)	0.714(3)	0.268 (4)	0.526(2)	0.338(3)	0.276(4)	-0.020 (9)	0.818(1)	0.335(3)
0.769(3)	0.808(3)	0.463 (3)	0.680(3)	0.536(3)	0.476(4)	0.0607 (4)	0.778(2)	0.428(3)
0.853(4)	0.837(4)	0.680 (4)	0.790(3)	0.696(4)	0.642(4)	0.0048 (3)	0.740(2)	0.501(3)
0.897(3)	0.910(3)	0.872 (3)	0.893(2)	0.844(3)	0.785(3)	-0.0084(2)	0.713(2)	0.570(3)

Table S4. **Measurements of bunching and full bunching probabilities, $p_{\mathbf{B}}$ and $p_{\mathbf{FB}}$, when only the polarization degree of freedom changes.** The triad phase φ, the three two-photon overlaps Δ_{12}, Δ_{23} and Δ_{31}, the average overlap $\bar{\Delta}$, the bunching and full bunching probability, p_B and p_{FB}, the Bargman invariant modulus $|\Delta_{123}|$, derived with two different methods, are presented here. These measurements are taken for different polarization states that belong to the plane that contains $|H\rangle$, $|V\rangle$, $|D\rangle$ and $|A\rangle$.

Δ_{12}	Δ_{23}	Δ_{31}	$\bar{\Delta}$	$\lvert\Delta_{123}^{V}\rvert$	$\lvert\Delta_{123}^{N}\rvert$	φ	p_B	p_{FB}
0.422(4)	0.902(3)	0.405(4)	0.576(3)	0.393(3)	0.355(8)	-0.01(1)	0.796(4)	0.366(7)
0.447(5)	0.861(4)	0.434(5)	0.581(4)	0.409(4)	0.342(9)	-0.03(2)	0.800(4)	0.362(8)
0.495(4)	0.900(3)	0.484(4)	0.626(3)	0.464(4)	0.397(9)	-0.02(1)	0.796(4)	0.394(7)
0.574(4)	0.874(3)	0.539(4)	0.662(3)	0.520(4)	0.454(8)	0.00(1)	0.780(4)	0.416(7)
0.644(4)	0.882(3)	0.596(4)	0.707(3)	0.582(4)	0.517(8)	0.005(9)	0.767(4)	0.445(7)
0.629(4)	0.894(3)	0.608(4)	0.710(3)	0.584(4)	0.519(8)	0.00 (1)	0.768(4)	0.448(7)
0.715(4)	0.892(4)	0.666(4)	0.758(3)	0.652(4)	0.589(8)	0.003 (8)	0.746(4)	0.472(7)
0.753(4)	0.878(3)	0.740(4)	0.790(3)	0.699(4)	0.650(7)	-0.006(7)	0.746(5)	0.512(7)
0.808(3)	0.892(3)	0.747(3)	0.815(3)	0.733(4)	0.674(6)	0.012(5)	0.736(4)	0.519(6)
0.886(3)	0.887(3)	0.847(3)	0.873(3)	0.816(4)	0.750(6)	0.0006(4)	0.723(4)	0.557(6)

Table S5. **Measurements of bunching and full bunching probabilities, $p_{\mathbf{B}}$ and $p_{\mathbf{FB}}$, when only the time degree of freedom changes.** The triad phase φ, the three two-photon overlaps Δ_{12}, Δ_{23} and Δ_{31}, the average overlap $\bar{\Delta}$, the bunching and full bunching probability, p_B and p_{FB}, the Bargman invariant modulus $|\Delta_{123}|$, derived with two different methods, are presented here. This measurements are taken for different time delay between one channel of the demultiplexer (DMX) and the other two.

Δ_{12}	Δ_{23}	Δ_{31}	$\bar{\Delta}$	$\lvert\Delta_{123}^{V}\rvert$	$\lvert\Delta_{123}^{N}\rvert$	φ	p_B	p_{FB}
0.264(6)	0.277(7)	0.315(6)	0.285(4)	0.152(4)	0.104(9)	-3.13(7)	0.889(3)	0.154(6)
0.268(7)	0.299(7)	0.287(7)	0.285(4)	0.152(4)	0.099(9)	-2.27(9)	0.883(3)	0.174(7)
0.317(6)	0.287(7)	0.299(7)	0.301(4)	0.165(4)	0.119(7)	-1.41(8)	0.845(3)	0.191(7)
0.315(6)	0.277(7)	0.314(6)	0.302(4)	0.165(4)	0.122(8)	-0.85(8)	0.831(4)	0.218(7)
0.286(6)	0.266(7)	0.240(7)	0.264(4)	0.135(4)	0.091(9)	0.015(8)	0.812(4)	0.206(7)
0.330(7)	0.283(7)	0.319(7)	0.311(4)	0.172(4)	0.109(9)	0.574(8)	0.819(4)	0.213(7)
0.302(7)	0.284(7)	0.367(7)	0.317(4)	0.177(4)	0.118(7)	1.25 (8)	0.839(4)	0.198(7)
0.290(7)	0.283(6)	0.276(7)	0.283(4)	0.151(4)	0.096(9)	2.17 (9)	0.869(3)	0.166(7)
0.291(7)	0.283(7)	0.303(6)	0.292(4)	0.158(4)	0.115(9)	2.73 (7)	0.894(3)	0.157(6)

Table S6. **Measurements of bunching and full bunching probabilities, $p_{\mathbf{B}}$ and $p_{\mathbf{FB}}$, when the triad phase φ changes by means of a liquid crystal.** The triad phase φ, the three two-photon overlaps Δ_{12}, Δ_{23} and Δ_{31}, the average overlap $\bar{\Delta}$, the bunching and full bunching probability, p_B and p_{FB}, the Bargmann invariant modulus $|\Delta_{123}|$, derived with two different methods, are presented here. These measurements are taken for different triad phases φ exploiting the liquid crystal.

depicted by the data presented in Tab. S6. Finally, data describing the counter-intuitive behavior of the full bunching probability p_{FB}, which initially increases and then decreases, modulated by the triad phase φ, as the average overlap $\bar{\Delta}$ increases, are presented in Tab. S7. The two-photon overlaps Δ_{12}, Δ_{23} and Δ_{31}, estimated via the measured visibility V_{ij} of independent Hong-Ou-Mandel experiments corrected for the non-zero multi-photon component estimated via

Δ_{12}	Δ_{23}	Δ_{31}	$\bar{\Delta}$	$\lvert\Delta_{123}^{\mathrm{V}}\rvert$	$\lvert\Delta_{123}^{\mathrm{N}}\rvert$	φ	p_{FB}
0.254(6)	0.061(6)	0.168(7)	0.161(4)	0.051(3)	0.023(2)	-0.23(9)	0.153(2)
0.255(6)	0.082(6)	0.169(6)	0.169(3)	0.059(3)	0.028(2)	-0.21(7)	0.158(2)
0.284(7)	0.102(6)	0.189(7)	0.192(4)	0.074(3)	0.040(3)	-0.18(5)	0.167(2)
0.291(6)	0.126(7)	0.198(7)	0.205(4)	0.085(3)	0.055(3)	-0.17(4)	0.177(2)
0.303(6)	0.185(6)	0.196(6)	0.228(4)	0.105(3)	0.072(3)	-0.12(3)	0.189(2)
0.274(6)	0.159(6)	0.261(6)	0.231(3)	0.107(3)	0.079(2)	-1.10(4)	0.185(2)
0.236(7)	0.239(6)	0.258(6)	0.244(4)	0.120(4)	0.094(3)	-3.63(2)	0.165(2)
0.254(6)	0.201(6)	0.326(6)	0.260(3)	0.129(4)	0.091(2)	-3.26(2)	0.157(1)

Table S7. **Measurements of counter-intuitive behaviour of the full bunching probability p_{FB} when $\bar{\Delta}$ increases.** The three two-photon overlaps Δ_{12}, Δ_{23} and Δ_{31}, the average overlap $\bar{\Delta}$, the modulus of the third-order Bargmann invariant $|\Delta_{123}|$, derived with two different methods, the full bunching probability p_{FB} and the triad phase φ are presented here.

$g^{(2)}(0)$ as in [17], are tabulated in all the tables in this section. With this independent measurement, one can estimate the modulus of the Bargmann invariant $|\Delta_{123}|$ corresponding to a given indistinguishability scenario in two different ways. First, $|\Delta_{123}^{\mathrm{V}}|$ can be derived from the measured two-photon overlaps as $\sqrt{\Delta_{12}\Delta_{23}\Delta_{13}}$; second, $|\Delta_{123}^{\mathrm{N}}|$ can be inferred from the experimentally measured photon number distributions with the analytical method presented in Supplementary Note 5. By these two independent estimations of the Bargmann invariant moduli we observe that $|\Delta_{123}^{\mathrm{N}}| < |\Delta_{123}^{\mathrm{V}}|$. This bias between the two different estimations can be explained by noting that the output photon number distribution is affected by the presence of multi-photon components, which are corrected in the estimation of $|\Delta_{123}^{\mathrm{V}}|$, and by the imperfect dialling of the unitary interferometer $\tilde{U}_3$, which slightly differs from an ideal balanced tritter matrix assumed in the derivation of Eq. (13).

Additionally, a complementary picture of the measured points in the main text section *"Experimental observation of counter-intuitive behavior of full bunching"* is shown in Fig. S1, where the measured Gram matrix preparations are depicted in the complex plane of the estimated third-order Bargmann invariant Δ_{123} for each experimentally measured configuration.

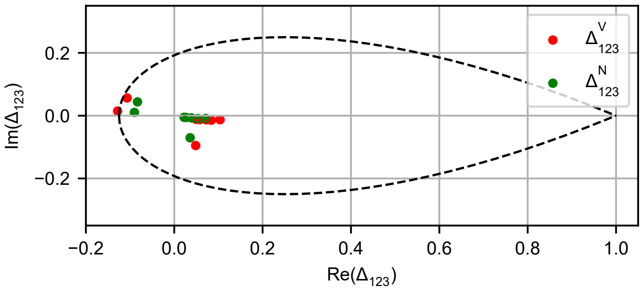

Figure S1. **Complex plane representation of the measured third-order Bargmann Invariants for the data in Table S7.** Behavior in the complex plane of the experimentally estimated Bargmann invariant parameters Δ_{123} for each Gram matrix preparation of Table S7. Red points correspond to the parameter $\Delta_{123}^{\mathrm{N}}$ estimated directly from the probability distributions, as highlighted in Supplementary Note 6. Conversely, green points correspond to the parameter $\Delta_{123}^{\mathrm{V}}$ as estimated via an independent measurement of the pairwise photon overlaps Δ_{ij} together with the triad phase φ from the former method. We also report with the black dashed line the border of the physically achievable values of Δ_{123} [2], as described in Supplementary Note 1.

As a complementary analysis to the observation of a counter-intuitive behaviour of the full-bunching probability, we note that while in the main text we consider p_{FB} as a function of the average pairwise overlap $\bar{\Delta}$, a similar behaviour holds also when the geometric mean of pairwise overlaps $|\Delta_{123}|^2$ is considered, as shown in Fig. S2. Indeed, when a balanced Fourier transformation is employed in a three-photon scenario, one can show that the full-bunching probability p_{FB} depends only on symmetric functions of the overlaps, namely the average overlap $\bar{\Delta}$ and the geometric mean $|\Delta_{123}|^2$, see Eqs. (4) and (5) of the main text. This joint analysis of Fig. 5 of the main text and Fig. S2 shows that even in the minimal non-trivial scenario, i.e. three-photons interfering in a three-mode interferometer, one can observe a counter-intuitive behaviour of the full bunching probability with respect to both the relevant symmetric functions of the pairwise overlaps by careful engineering of indistinguishability scenarios involving both polarization

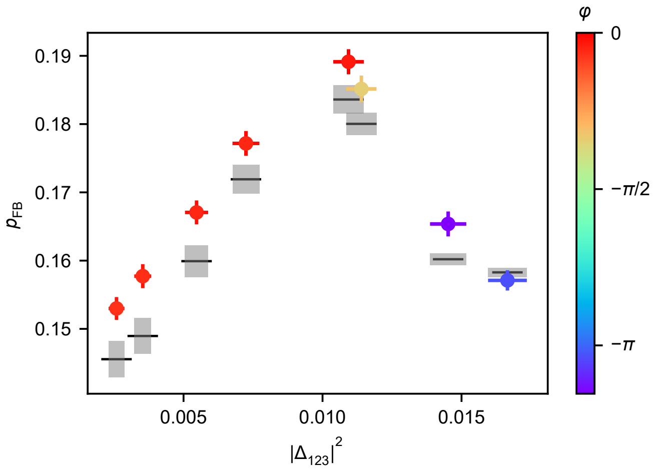

Figure S2. **Counter-intuitive behaviour of full bunching probabilities -** We report the measured full-bunching probabilities p_{FB} measured experimentally at the output of a balanced Fourier interferometer as a function of the geometric mean $|\Delta_{123}|^2$ computed from the measured pairwise overlaps as $|\Delta_{123}|^2 = \Delta_{12}\Delta_{23}\Delta_{13}$. The colormap shows the associated triad phase $\varphi = \arg(\Delta_{123})$, while the grey boxes identify the theoretical expectation simulated numerically considering the experimentally measured Gram matrices. Again, we observe that one can have a decreasing full-bunching probability even if the geometric mean of pairwise overlaps $|\Delta_{123}|^2$ increases.

and time degrees of freedom.

Supplementary References

[1] Horn, R. A. & Johnson, C. R. Matrix analysis. (Cambridge: Cambridge university press, 2012).
[2] Fernandes, C. et al. Unitary-invariant witnesses of quantum imaginarity. *Physical Review Letters* **133**, 190201 (2024).
[3] Wanless, I. M. Permanents of matrices of signed ones. *Linear and Multilinear Algebra* **53**, 427-433 (2005).
[4] Heindel, T. et al. Quantum dots for photonic quantum information technology. *Advances in Optics and Photonics* **15**, 613-738 (2023).
[5] Senellart, P., Solomon, G. & White, A. High-performance semiconductor quantum-dot single-photon sources. *Nature Nanotechnology* **12**, 1026-1039 (2017).
[6] Gazzano, O. et al. Bright solid-state sources of indistinguishable single photons. *Nature Communications* **4**, 1425 (2013).
[7] Somaschi, N. et al. Near-optimal single-photon sources in the solid state. *Nature Photonics* **10**, 340-345 (2016).
[8] Ollivier, H. et al. Reproducibility of high-performance quantum dot single-photon sources. *ACS Photonics* **7**, 1050-1059 (2020).
[9] Thomas, S. et al. Bright polarized single-photon source based on a linear dipole. *Physical Review Letters* **126**, 233601 (2021).
[10] Hong, C. K., Ou, Z. Y. & Mandel, L. Measurement of subpicosecond time intervals between two photons by interference. *Physical Review Letters* **59**, 2044-2046 (1987).
[11] Antón, C. et al. Interfacing scalable photonic platforms: solid-state based multi-photon interference in a reconfigurable glass chip. *Optica* **6**, 1471-1477 (2019).
[12] Pont, M. et al. Quantifying n-photon indistinguishability with a cyclic integrated interferometer. *Physical Review X* **12**, 031033 (2022).
[13] Pont, M. et al. High-fidelity four-photon GHZ states on chip. *npj Quantum Information* **10**, 50 (2024).
[14] Rodari, G. et al. Semi-device-independent characterization of multiphoton indistinguishability. *PRX Quantum* **6**, 020340 (2025).
[15] Clements, W. R. et al. Optimal design for universal multiport interferometers. *Optica* **3**, 1460-1465 (2016).
[16] Flamini, F. et al. Thermally reconfigurable quantum photonic circuits at telecom wavelength by femtosecond laser micromachining. *Light: Science & Applications* **4**, e354 (2015).
[17] Ollivier, H. et al. Hong-ou-mandel interference with imperfect single photon sources. *Physical Review Letters* **126**, 063602 (2021).
[18] Oszmaniec, M. & Brod, D. J. Classical simulation of photonic linear optics with lost particles. *New Journal of Physics* **20**, 092002 (2018).
[19] Menssen, A. J. et al. Distinguishability and many-particle interference. *Physical Review Letters* **118**, 153603 (2017).
